# Practical Considerations for Bedside Teaching: A Critical Application of Kolb's Experiential Learning Cycle

**DOI:** 10.1111/tct.70437

**Published:** 2026-04-30

**Authors:** Raabia Farooqi, Diane Scutt

**Affiliations:** ^1^ Manchester University NHS Foundation Trust Manchester UK; ^2^ Edge Hill University Ormskirk UK; ^3^ Division of Musculoskeletal and Dermatological Science, Faculty of Biology, Medicine and Health University of Manchester Manchester UK

## Abstract

Bedside teaching represents an immensely valuable learning experience for students, fostering acquisition of clinical skills alongside wider professional competencies. Despite such benefits, the practice of bedside teaching is in decline, and there remains a paucity of available guidance regarding how best to conduct bedside teaching. Learning theories can enhance teaching through providing evidence‐based frameworks; Kolb's experiential learning theory is of particular relevance to bedside teaching due to its immersive and hands‐on nature. This article critically applies experiential theory to bedside teaching and derives relevant considerations for tutors. It is found that bedside teaching is rooted in and evidenced by experiential learning theory and should thus be prioritised within clinical placements. Experiential theory can be used to optimise the learning experience provided, structure learning within clinical contexts and emphasise reflection and subsequent student learning. However, there is a need for dynamic adaptation of the model in response to unpredictable environments and for tutors to support and guide students through the learning cycle. Further empirical research is required to establish the efficacy of direct employment of Kolb's learning cycle within bedside teaching and to optimise the concrete experience provided.

## 1 Introduction

Bedside teaching epitomises the famous statement by Sir William Osler that ‘Medicine is learned by the bedside and not in the classroom’ [1]. By definition, bedside teaching is active learning that takes place in the presence of a patient within the clinical environment. This learning usually takes place within a small group at the patient's bedside and comprises clinical activities including history‐taking and examination involving the triad of patient, learner and tutor [2]. Through hands‐on, active learning, bedside teaching enhances clinical competence as well as wider professional capabilities such as communication, empathy and patient‐centred practice [3].

Despite the established benefits of bedside teaching, literature suggests that the practice is declining due to factors including introduction of novel healthcare technologies and increased patient turnover [4]. Furthermore, there is a paucity of relevant guidance pertaining to conducting bedside teaching; for instance, a review of medical education textbooks found that very few provided significant information on bedside teaching [5]. Given the aforementioned advantages and indication of recent neglect within clinical practice, it appears there is a compelling need to further establish how best to conduct bedside teaching to maximise benefit for students and to preserve its implementation within medical education.

*There is a compelling need to further establish how best to conduct bedside teaching to maximise benefit for students and to preserve its implementation within medical education.*

Teaching and learning theories are of pivotal importance to provide evidence‐based frameworks for teaching, enhance the organisation and quality of learning produced and increase motivation and well‐being of students [6]. As such, application of learning theory to bedside teaching can inform and evidence practices. Experiential learning theory has particular pertinence to the methodology and ethos of bedside teaching due to its situation within the clinical context and focus on learning through experience.

Within this article, experiential learning theory is critically applied to bedside teaching to establish the benefits of bedside teaching within its framework, its value and limitations within this context and how it can offer an understanding of relevant considerations when conducting bedside teaching. Practical applications are derived that may be used by educators to inform and enhance bedside teaching practice.

## 2 Experiential Learning and Bedside Teaching

Experiential learning theory posits that learning is ‘the process whereby knowledge is created through the transformation of experience’ [7]. This concept is also central to Knowles' theory of andragogy, which poses that experiences accumulated by adult‐learners form a rich learning resource [8]. The most widely established model underpinning experiential theory is Kolb's four‐stage learning cycle, which proposes that learners must undergo *concrete experience*, subsequent *reflective observation*, and then derive learning through *abstract conceptualisation*. Following this, the learner undertakes *active experimentation*, which in turn forms a further *concrete experience* [7].

**FIGURE 1:**
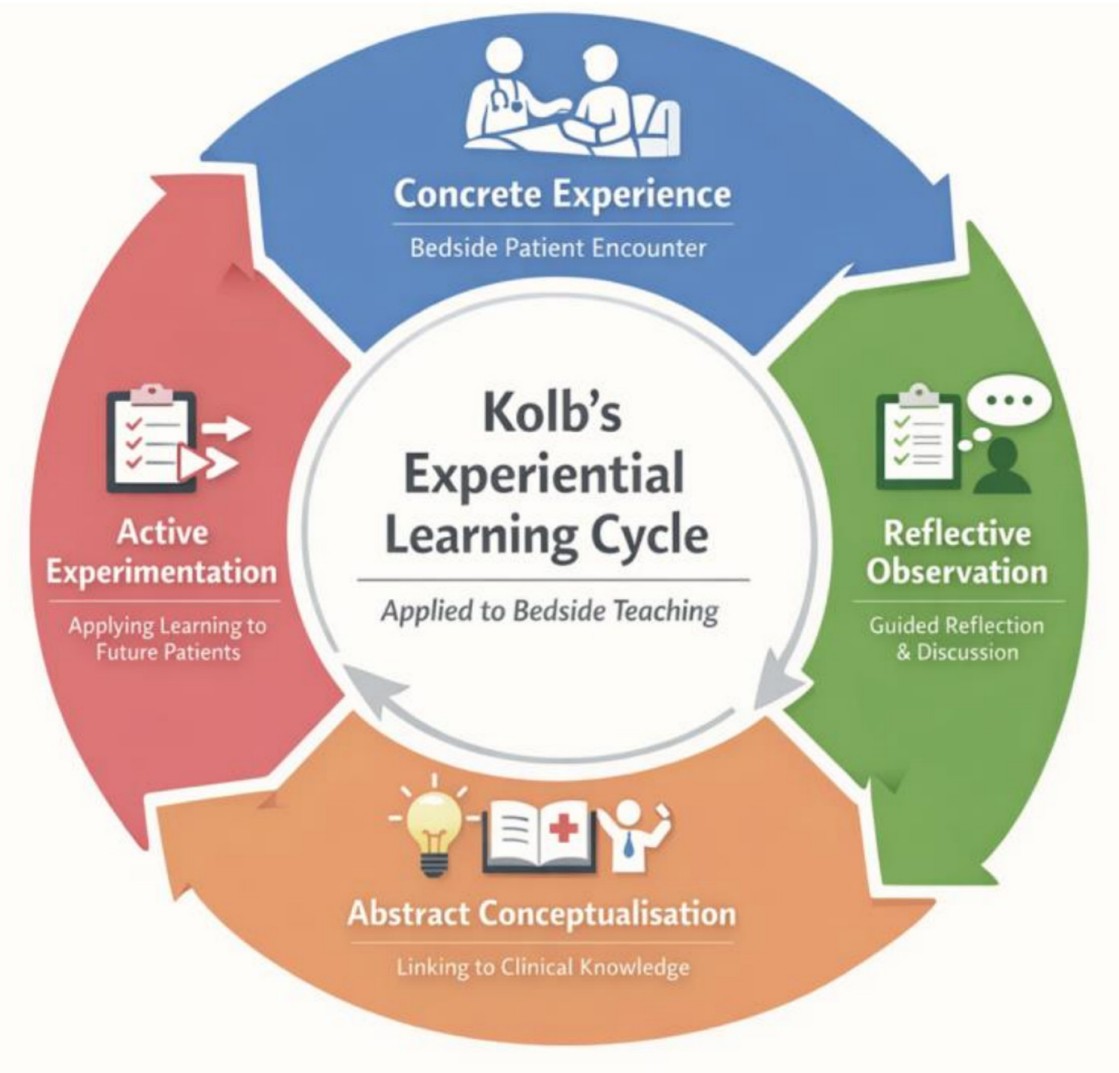
Application of Kolb's experiential learning cycle to bedside teaching.

As bedside teaching operates on the premise of learning directly from clinical exposure, the application of experiential theory is inherently of relevance and utility. One invaluable aspect of bedside teaching is that it is situated directly within the clinical environment at the patient's bedside; this extends learning far beyond solely clinical skills, as it cultivates crucial professional capabilities such as resilience, the capacity to respond to unpredictability and ability to conduct oneself within the doctor‐patient encounter. This holistic benefit of ‘real‐life’ education is central to experiential learning, which situates the workplace as a rich learning environment to supplement formal education, and establishes the linkage between education, work and personal development [7]. Literature substantiates that experiential learning models can foster positive student behaviours including enhanced clinical decision‐making, confidence and patient communication [9]. As such, it may be concluded that bedside teaching is rooted within and evidenced by experiential theory, and its usage can facilitate diverse skill acquisition within clinical contexts. Figure 1 further illustrates the applicability of Kolb's cycle directly to bedside teaching.

*Beside teaching is rooted within and evidenced by experiential theory, and its usage can facilitate diverse skill acquisition within clinical contexts.*

We now discuss literature pertaining to experiential learning and consider how its principles can be applied to optimise bedside teaching in practice. In particular, we explore how the quality of the concrete experience may be enhanced, how structured reflection and conceptualisation can be effectively facilitated within clinical environments and how educators can support learners in progressing through the stages of Kolb's cycle. We thus aim to translate experiential learning theory into practical strategies that can be readily implemented by educators.

## 3 Optimising the Concrete Experience

Central to experiential theory is the ideology of ‘learning through doing’, which is also a crucial tenet of bedside teaching. Evidence consistently supports that students engage more and retain greater knowledge through active participation as opposed to lecture‐based teaching; this is tangibly illustrated by trials demonstrating enhanced performance among medical students assigned to experiential learning programmes as opposed to more traditional, didactic pedagogy [10]. According to experiential theory, such learning can be achieved as clinical activities form concrete experiences from which the student can subsequently derive learning through the learning cycle [7]. However, it remains that Kolb's theory has faced criticism due to a lack of clarity surrounding what exactly constitutes a concrete experience, which has been deemed as ‘muddled’ by critics [11]. As such, it seems that considerations regarding the quality and nature of experience provided to learners are needed.

A recent systematic review critiquing Kolb's cycle found that concrete experiences may be optimised through promoting learner responsibility within the learning process [12]. Such considerations could be applied to enhance the concrete experience offered by bedside teaching. Learner responsibility may be promoted by allowing supervised independence during history‐taking and clinical examinations, with tutors intervening only when necessary to ensure patient safety and guide the interaction. Furthermore, within the context of small‐group bedside teaching, assigning defined roles allows students to take ownership of specific components of the encounter; this may include leading the history‐taking, performing the focused examination, observing communication and summarising clinical findings. Such measures can transform learners from passive observers into active participants, thereby engaging participation through learner responsibility and accountability and enhancing the learning attained from the concrete experience.

Learner responsibility can further be encouraged by conducting structured discussion following the bedside teaching activity, prompting autonomous clinical decision‐making by learners. This may include asking students to formulate differential diagnoses, propose investigations and devise initial management plans prior to tutor input. Such questioning requires learners to articulate and justify their clinical reasoning, thereby supporting deeper learning and diagnostic thinking. However, it remains that such independence must be balanced with students' confidence and experience levels; early learners may benefit from greater scaffolding, through prompting with questions or structured frameworks for clinical reasoning, whereas more advanced learners can be encouraged to manage larger components of the encounter independently [5]. By progressively increasing learner autonomy while maintaining appropriate supervision, educators can support competence development while maintaining learner confidence during bedside teaching.

Furthermore, Morris [12] found in their systematic review that providing contextual richness is important to enhance learning from the concrete experience. Within bedside teaching, discussion of pertinent clinical, sociocultural and workplace factors can help situate learning within the broader clinical environment and encourage a more holistic clinical approach. In practical terms, tutors may ask learners to identify relevant risk factors from the history, consider how the patient's social circumstances and demographics influence their health behaviours and explore how the patient's own understanding of their condition shapes communication during the encounter, thereby prompting discussion of the influence of wider factors on a patient's overall presentation and management. In addition to patient‐related factors, workplace dynamics can also provide valuable learning context to bedside teaching; tutors may therefore invite reflection during debriefing on how environmental factors influenced the interaction, such as the presence of family members, interruptions from clinical staff or time pressures within the ward environment. Discussing such contextual influences facilitates provision a more holistic experience that is synergetic with the healthcare environment, enhancing the learning attained.

A further important means of contextualisation within bedside teaching is situating the current encounter within students' prior experiences, facilitating integration of new knowledge with existing understanding. Rotthoff [13] further highlights the value of conducting a purposeful briefing prior to bedside teaching to activate learners' existing knowledge and appropriate contextualisation of the current experience. Tutors may ask students about their pre‐existing experiences with history‐taking and examinations, and any learning from previous sessions, thereby facilitating active experimentation with new knowledge. This also readily prepares learners to engage with the session. Such briefing also allows educators to gauge learners' baseline knowledge and tailor the encounter accordingly, ensuring that the bedside teaching experience is appropriately challenging and educationally meaningful.

Another pertinent consideration to contextualisation of the concrete experience is assigning attention to learners' emotional readiness and orientation to the clinical environment, particularly for early clinical learners. Brief orientation to the ward environment, equipment and team members can enhance learners' confidence and engagement, supporting active participation within the clinical setting [14]. Tutors should also consider addressing learners' anxieties prior to patient interaction, for example, by inviting discussion of concerns about communication or clinical performance; normalising such uncertainty can help establish psychological safety, which permits interpersonal risk‐taking [15]. This importance of actively engaging and responding to learner emotions within experiential learning has been elucidated in literature, serving to heighten the learning and memory response and channel negative emotions to deeper reflective learning [16]. Creating a supportive and psychologically safe environment through purposeful briefing therefore enables learners to engage more fully with the clinical encounter, strengthening the quality of the concrete experience.

Therefore, although bedside teaching provides an ideal concrete experience for experiential learning, care should be taken by tutors ensure that the experience provided is of a high quality. This may be enhanced through purposeful contextualisation of the encounter, integration of prior knowledge and by promoting learner autonomy and responsibility throughout the activity.

## 4 Providing Structure Within the Clinical Environment

An advantageous application of Kolb's learning cycle to bedside teaching is provision of a sound structure to learning. The clinical environment is complex and highly unpredictable, which can create unique challenges for learning [17]. Indeed, bedside teaching may easily become disrupted by workplace pressures including emergency tasks and conflicting patient activities, which may cause neglection of proper debriefing afterwards. Employment of Kolb's cycle within this context is immensely beneficial to maintain a structured approach, ensuring that crucial stages of reflection and conceptualisation are not overlooked and can be revisited. In practice, Kolb's cycle can act as a cognitive checklist for educators, ensuring that learning does not conclude prematurely at the level of the experience alone; even where interruptions occur, the framework enables tutors to return to missed stages, for instance, by scheduling a subsequent postencounter debrief, thereby preserving educational coherence despite clinical unpredictability.

However, whereas this provision of a core structure may be immensely valuable, Kolb's learning cycle has faced criticism for its linear process, which may not emulate real‐life experience, and could be too simplistic for use within the complexities of modern workplaces [18]. Such limitations are highly applicable within the clinical environment, in which unforeseen factors pertaining to the workplace, patient and student inevitably arise and must be adapted for; for instance, the scenario may be paused midway due to student or patient concerns, or reflection and conceptualisation may occur during the experience itself to problem‐solve, or active experimentation may take place straight away in response to feedback during the bedside teaching experience. This dynamic nature and need for rapid adaptation can necessitate jumping through stages in the cycle rather than following them sequentially.

In practical terms, tutors should aim to apply Kolb's cycle as a flexible scaffold rather than a rigid sequence, with dynamic adaptation in accordance with student needs, the developing clinical environment and external factors. This may involve incorporating brief ‘reflection‐in‐action’ prompts during the encounter if students become stuck (for instance, ‘what are you thinking at this stage?’ or ‘what would you do next?’) to facilitate immediate conceptualisation and experimentation with appropriate tutor guidance. Furthermore, missed stages should be revisited and addressed afterwards to ensure that learning can be guided through the experiential learning cycle.

*Tutors should aim to apply Kolb’s cycle as a flexible scaffold rather than a rigid sequence, with dynamic adaptation in accordance with student needs and the developing clinical environment.*

Overall, Kolb's cycle offers an excellent basis for structuring bedside teaching and ensuring thorough reflection; however, the dynamic nature of clinical environments may impede sequential employment of the model, and there is a need for educators to adapt to the evolving scenario and the needs of individual learners.

## 5 Encouraging Reflective Practice Through Structured Debriefing

A further beneficial application of experiential theory to bedside teaching is the emphasis upon reflection, which comprises an important stage of the learning cycle. Reflection is a crucial professional skill within clinical practice; experiential learning facilitates this development and significantly improves reflective and self‐evaluative capabilities among medical students [19]. Within bedside teaching, reflection can be effectively facilitated through structured debriefing following the patient encounter.

Debriefing may be conducted away from the immediate clinical environment to optimise reflection and allow physical distance from any stress experienced during the clinical encounter; this approach is reinforced by embodied learning theory, which posits that sociospatial interaction of the body with the environment contributes profoundly to learning and perception [20]. Broadly, debriefing should aim to guide students through the reflective observation and abstract conceptualisation of Kolb's cycle, enhancing preparedness for subsequent active experimentation during future encounters. Tutors may implement a simple structured framework such as the Pendleton model for initial feedback to prompt reflection, encouraging learners to identify what went well and areas for improvement before providing tutor input [21]. This ensures that reflective observation is student‐led, which is central to Kolb's own theory [7], and allows subsequent provision of highly tailored feedback based on points raised by the student. Tutors should then aim to transform reflective observation into abstract conceptualisation through linking the reflective observations drawn to pertinent clinical principles and frameworks to consolidate learning and inviting students to share what learning they will take forward for next time for further active experimentation.

It is also important to recognise that heterogeneity in learners' capacity and motivation for reflection can significantly limit learning attained from reflective learning; this is reaffirmed by literature that establishes that poor learner engagement and metacognitive limitations can impede reflection [22]. This can be addressed through guided reflection in debriefing, with tutors providing prompts and support to scaffold learners who may struggle to reflect independently and require further guidance as opposed to reflection being purely student‐led. To ensure active participation of all learners in reflective observation, tutors may adopt strategies such as directed questioning, role allocation and inviting each student to contribute a single observation or learning point; such approaches help prevent passive observation and promote inclusive engagement.

Furthermore, literature demonstrates that tutor‐led debriefing can be a valuable method to ensure that conceptualisations drawn by students align with the educator's learning goals for the activity and to rectify any student misinterpretations [23]. This is a crucial measure to ensure that student‐led reflection and conceptualisation are correct and appropriate. In practice, tutors may explicitly revisit the session's goal during the debriefing so that students may align their learning accordingly (for instance, ‘we aimed to practice cardiovascular examinations during this session. What did you learn about how to perform this effectively today?’). Misconceptions that arise during discussion should be corrected by tutors through guiding learners to reconsider their reasoning. Tutors may also summarise key take‐home points at the end of the discussion to consolidate accurate learning.

Reflection and debriefing therefore represent important applications of experiential learning theory to bedside teaching, with holistic educational and professional benefit for students; care must be taken that reflection is student‐led, transformed into conceptualisation and guided by tutors to align with educational goals and curricula.

## 6 Practical Challenges and Limitations of Bedside Teaching

Although bedside teaching offers substantial educational value, pertinent practical limitations must be acknowledged. Effective bedside teaching typically requires small group sizes to enable active participation and meaningful supervision, which may be difficult to achieve within time‐pressured clinical environments [2]. In addition, repeated involvement of patients in teaching may contribute to fatigue, and there is potential for discomfort or distress when sensitive topics are explored at the bedside. The unpredictability of clinical settings, including interruptions and competing service demands, may further limit opportunities for structured teaching and reflection.

In practice, such challenges necessitate a flexible and patient‐centred approach. Tutors should seek to minimise patient burden by limiting the duration of bedside interactions, obtaining informed consent and establishing any topics the patient would prefer to avoid. Where appropriate, more sensitive discussions or extended teaching can be conducted away from the bedside. Careful attention to timing, including avoiding ward rounds or mealtimes, can enhance both patient experience and learner engagement through minimising disruption. Thus, a pragmatic approach that balances experiential learning and educational objectives with patient wellbeing and clinical realities is therefore required to ensure that bedside teaching remains both effective and sustainable.

*A pragmatic approach that balances experiential learning and educational objectives with patient wellbeing and clinical realities is therefore required.*

## 7 Practical Applications and Considerations of Experiential Learning Theory Within Bedside Teaching

**FIGURE 2:**
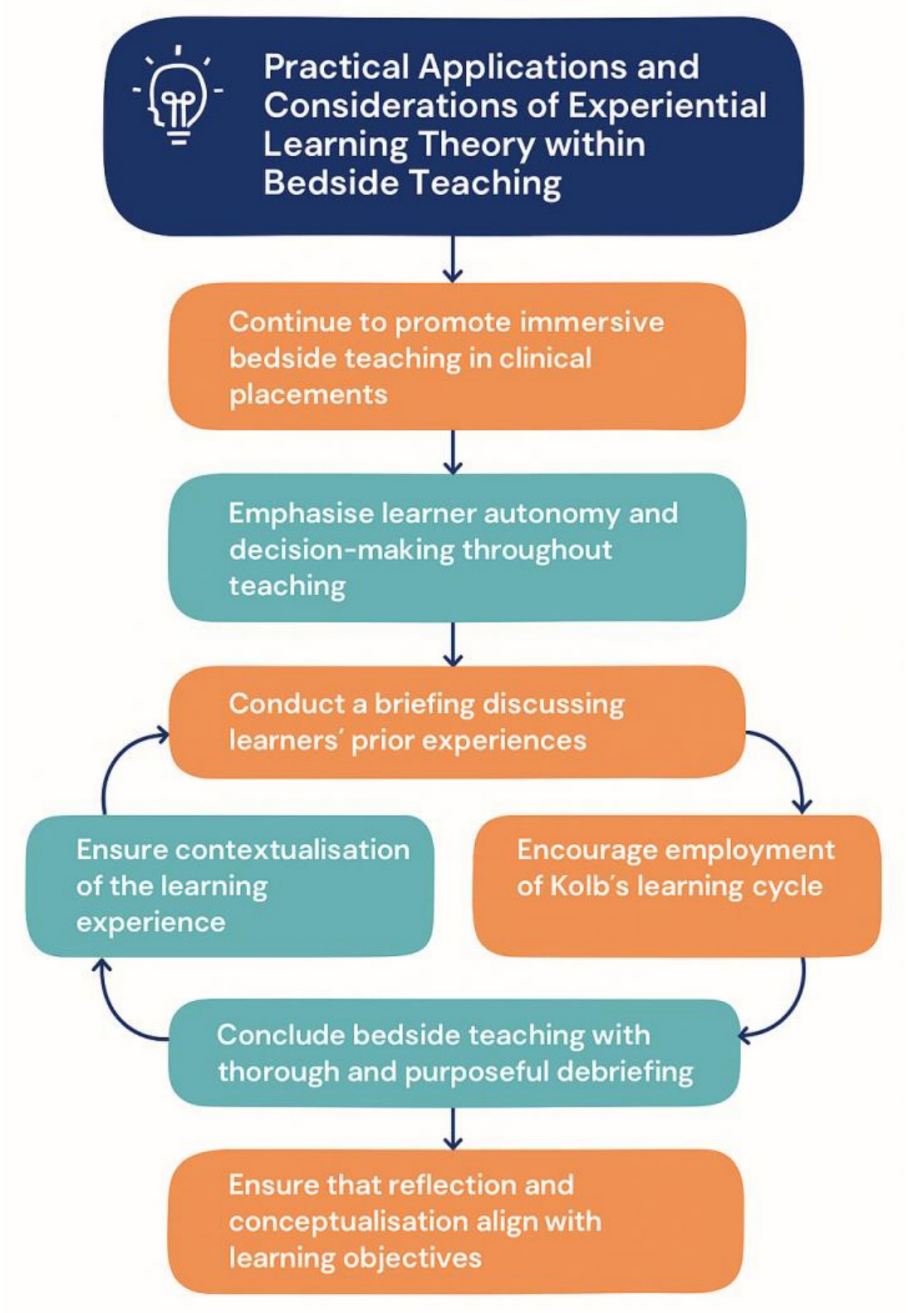
Practical considerations for bedside teaching.

Further to the above discussion, we derive practical considerations for the application of experiential learning theory within bedside teaching, which is further summarised within Figure 2.

1. Promote bedside teaching within clinical placements to provide immersive, authentic learning experiences that support the development of clinical competence alongside wider professional skills, in keeping with the principles of experiential learning.
2. Emphasise learner autonomy and responsibility throughout the bedside encounter to optimise the concrete experience. This may include allowing supervised independence in history‐taking and examination; encouraging learners to generate differential diagnoses, investigations and management plans; and assigning defined roles to promote active participation.
3. Conduct a purposeful prebriefing prior to the session to activate learners' prior knowledge, clarify expectations and support integration of new experiences with existing understanding, thereby facilitating progression through the experiential learning cycle.
4. Ensure adequate contextualisation of the clinical encounter by incorporating discussion of patient‐related, sociocultural and workplace factors. This includes consideration of patient demographics, lifestyle influences, clinical journey and environmental dynamics to promote holistic understanding and enhance the richness of the learning experience.
5. Attend to learners' emotional readiness and establish psychological safety by acknowledging anxieties, normalising uncertainty and fostering a supportive learning environment, particularly for early clinical learners, to enhance engagement with the bedside encounter.
6. Adapt supervision according to learner experience through appropriate scaffolding, providing guidance and structured support for less experienced learners while enabling greater independence for more advanced learners to promote confidence and competence development.
7. Apply Kolb's experiential learning cycle as a flexible scaffold to structure bedside teaching, ensuring progression through experience, reflection, conceptualisation and application, while adapting dynamically to the clinical environment, patient factors and learner needs.
8. Conclude bedside teaching with structured debriefing to facilitate reflective observation and abstract conceptualisation. This may incorporate established frameworks such as Pendleton's model to promote learner‐led reflection and targeted feedback.
9. Ensure active participation of all learners during debriefing through inclusive strategies such as directed questioning or structured turn‐taking, with additional support provided to learners who may require guidance in reflective practice.
10. Use debriefing to align learning with intended objectives and address misconceptions, by revisiting session goals, reinforcing key clinical principles and ensuring that learners' conceptualisations are accurate and educationally meaningful.

**FIGURE 3:**
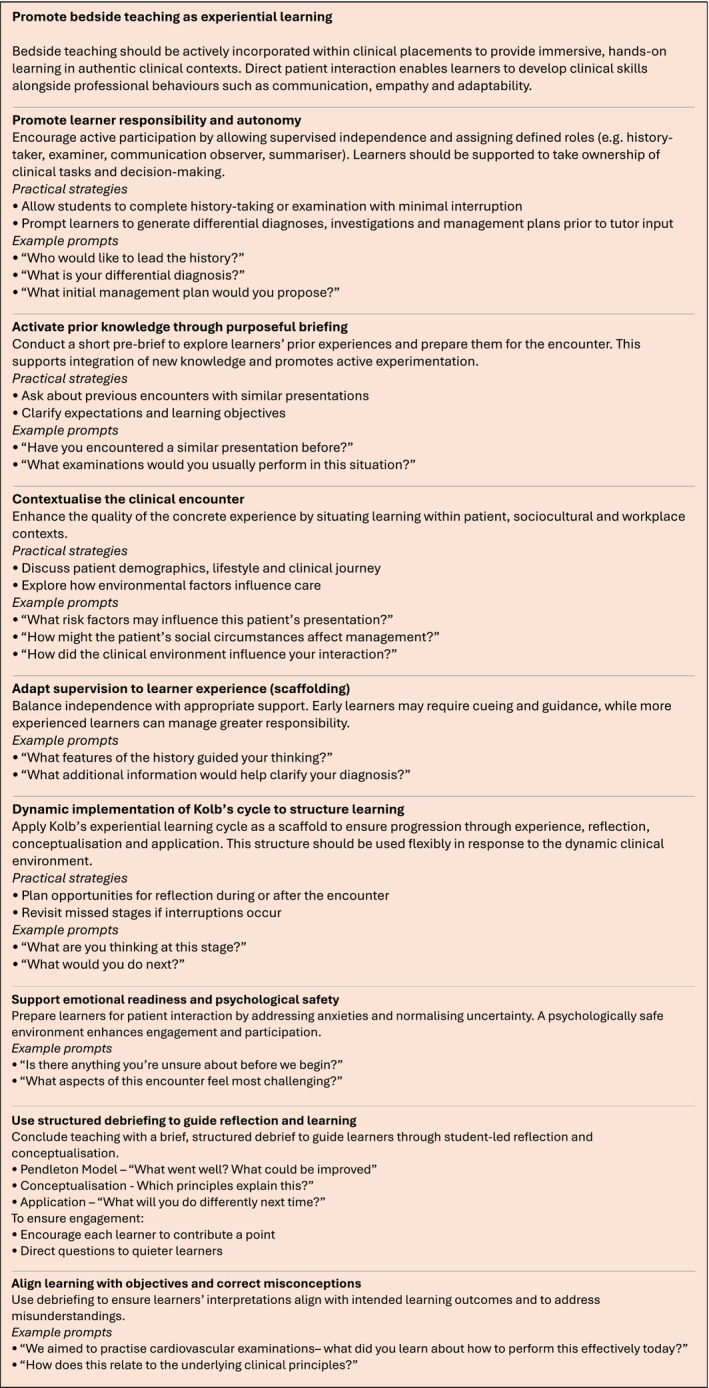
A summary of practical guidance to improve bedside teaching with example prompts.

Figure 3 further provides practical considerations and example prompts to aid in translating the points above directly into teaching practice.

## 8 Conclusion

Experiential learning theory evidences bedside teaching and provides important guidance on structuring reflection, growth and learning from the experience. Bedside teaching benefits from ‘real‐life’, contextually rich teaching involving active, hands‐on learning; these principles are firmly rooted within experiential learning theory and enable acquisition of wider professional capabilities. Despite such benefits, the practice of bedside teaching is declining, and there is a compelling need to emphasise its use within medical education.

Application of Kolb's cycle has the potential to improve structured learning within the clinical environment. However, educators must also understand the need for dynamic adaptation of the model when applied to bedside teaching. It may therefore be proposed that facilitators of bedside teaching should be trained in the principles of experiential learning theory, with emphasis on adoption of a flexible and integrated approach. Experiential learning further emphasises the need for reflection to achieve learning, which is highly applicable to bedside teaching. This may be best achieved through debriefing to guide students through purposeful reflection in alignment with educational curricula, allowing educators to gauge the learning attained. There is a need to better understand how to optimise bedside teaching as a ‘concrete experience’ for students; although considerations such as contextualisation and learner autonomy can improve experiential learning models, further educational research is needed regarding measures that can enhance the experience provided by bedside teaching specifically.

## Author Contributions

**Raabia Farooqi:** conceptualization, investigation, methodology, project administration, writing – original draft, writing – review and editing. **Diane Scutt:** conceptualization, supervision, writing – review and editing.

## Funding

The authors have nothing to report.

## Conflicts of Interest

The authors declare no conflicts of interest.

## Data Availability

Data sharing is not applicable to this article, as no datasets were generated or analysed during the current study.
